# The 1 % of the population accountable for 63 % of all violent crime convictions

**DOI:** 10.1007/s00127-013-0783-y

**Published:** 2013-10-31

**Authors:** Örjan Falk, Märta Wallinius, Sebastian Lundström, Thomas Frisell, Henrik Anckarsäter, Nóra Kerekes

**Affiliations:** 1 CELAM (Centre for Ethics, Law and Mental Health), Institute of Neuroscience and Physiology, University of Gothenburg, Wallinsgatan 8 Plan 5, 431 41 Mölndal, Sweden; 2 Department of Clinical Sciences Malmö, Lund University, Lund, Sweden; 3 Clinical Epidemiology Unit, Department of Medicine, Karolinska Institutet, Stockholm, Sweden; 4 R&D Unit Gothenburg, Swedish Prison and Probation Service, Gothenburg, Sweden

**Keywords:** Persistent offender, Violent crime, Population based, Nationwide registry, Mental health

## Abstract

**Purpose:**

Population-based studies on violent crime and background factors may provide an understanding of the relationships between susceptibility factors and crime. We aimed to determine the distribution of violent crime convictions in the Swedish population 1973–2004 and to identify criminal, academic, parental, and psychiatric risk factors for persistence in violent crime.

**Method:**

The nationwide multi-generation register was used with many other linked nationwide registers to select participants. All individuals born in 1958–1980 (2,393,765 individuals) were included. Persistent violent offenders (those with a lifetime history of three or more violent crime convictions) were compared with individuals having one or two such convictions, and to matched non-offenders. Independent variables were gender, age of first conviction for a violent crime, nonviolent crime convictions, and diagnoses for major mental disorders, personality disorders, and substance use disorders.

**Results:**

A total of 93,642 individuals (3.9 %) had at least one violent conviction. The distribution of convictions was highly skewed; 24,342 persistent violent offenders (1.0 % of the total population) accounted for 63.2 % of all convictions. Persistence in violence was associated with male sex (OR 2.5), personality disorder (OR 2.3), violent crime conviction before age 19 (OR 2.0), drug-related offenses (OR 1.9), nonviolent criminality (OR 1.9), substance use disorder (OR 1.9), and major mental disorder (OR 1.3).

**Conclusions:**

The majority of violent crimes are perpetrated by a small number of persistent violent offenders, typically males, characterized by early onset of violent criminality, substance abuse, personality disorders, and nonviolent criminality.

## Introduction

Interpersonal violence remains one of the leading causes of impaired quality of life and mortality in the world, especially among people aged 15–44 years. In 2000, homicides accounted for half a million deaths worldwide; almost twice as many as in wars during the same year [1]. The global costs of violent crimes, both in terms of economy and human suffering, are massive, and the impact of violence on worldwide insecurity, disability, and mortality is predicted to increase in the coming decades [2]. The World Health Organization (WHO) has stated that the prevention of violence must be a global public health priority [1].

In order to establish specific treatments and preventive strategies, it is crucial to have a detailed understanding of the distribution of violent crimes across first time offenders and recidivists at different stages of their violent careers. It is also necessary to distinguish between risk for single-episode violent crime and for the development of persistent violence in individuals. That comparatively few individuals present with disinhibitory behaviors very early in life, go on to a career of “life-course persistent antisocial behavior,” and will ultimately account for more than half of all violent crimes, and an even larger proportion of aggravated crimes, were presented by Moffit and Caspi using (among others) data-sets from the Dunedin birth cohort studies [3–5]. These results are important for the description of the development of persistently violent individuals.

General prevention may be directed at violence per se, but specific treatment must be directed to individuals who have demonstrated their propensity for violence through repeat offenses, as this is currently the only certain way the most violence-prone individuals present themselves to jurisprudence. A scientific rationale for such efforts requires a detailed understanding of the role of persistence in the total crime figures, as well as the identification of risk factors specifically related to persistence in criminal violence. Even if it is accepted that the majority of violent crimes are committed by individuals who have previously been convicted for several crimes (any kind of offense) [6], and that recidivism rates after juvenile detention and prison are high [7, 8], few studies have tried to quantify the burden of violent crime in the total population, and most previous research has focused on risk factors of ever having been convicted of a violent crime [9, 10] rather than the risk for relapsing. For clinicians, the specific challenge is to prevent further recidivism in patients who have already developed a pattern of violence, as it is much more difficult to identify individuals at need for preventive treatment before the violent behavior has presented itself.

Among known risk factors for being convicted of a violent crime, male sex is the most prominent; men commit about 90 % of violent crimes [1, 3]. Substance abuse carries an increased risk for violent crime, both among offenders [11] and in general population samples [12–14]. Antisocial personality disorder is another risk factor for violence [9, 15], although individuals diagnosed with major mental disorders have an overall higher risk than the general population for criminal (including violent) offending [16–19], a large part of this increased risk seems to be attributable to concomitant substance abuse [20–22].

Previous criminal and aggressive behaviors have consistently been identified as the best predictor for new crimes [23, 24]; yet our current knowledge about individuals who persist in criminal behavior and possibilities for treatment remains sketchy at best.

In the present study, a Swedish population-based research database with interlinked conviction data on all types of crime was used to determine the distribution of violent convictions in the total Swedish population, 1973–2004. School grades, psychiatric diagnoses, and parental characteristics from official nationwide registers were included in the database to identify criminal, educational, parental, and psychiatric risk factors for persistence in violent crime (defined as a history of three or more convictions for violent crimes). The specific aims for the analyses were to:

1.
  - map the distribution of violent crime convictions,
  - identify gender differences,
  - characterize the subgroup responsible for the majority of all violent crime convictions, and
2. identify criminal, educational, parental, and psychiatric risk factors for persistence in violent crime versus having one or two such convictions.

## Methods

### Ethics statement

The Research Ethics Committee at Karolinska Institutet approved the study, DNR 521-2010-2689.

### Register data

The unique personal identification number that is ascribed to each Swedish citizen upon birth or arrival to the country was used to merge data across nationwide registries [15, 25]. Data were anonymized in the linkage process by the transformation of the personal identification number into a randomly assigned coded string variable. In this study, the total follow-up period spans from 1973 to 2004, and includes summary information on parental data prior to 1973. The registers used were Crime Register, Total Population Register, Cause of Death Registry and Migration Registers, Compulsory 9-year Comprehensive School Register, and Hospital Discharge Register [26]. A working data file for the present study was created including variables chosen to investigate the relationship between mental health factors and persistence in violent criminality.

The Crime Register (National Council of Crime Prevention) contains records of all convictions in Swedish lower courts from January 1, 1973 to December 31, 2010, including custodial and noncustodial (i.e., probation and/or fines) sentences. In Sweden, offenders cannot be considered “not guilty by reason of insanity.” Individuals who commit crimes under the influence of a severe mental disorder are generally sentenced to compulsory forensic psychiatric care. Anyone charged with a crime is tried in court and, if convicted, entered into the official Crime Register. Criminal responsibility begins at age 15 in Sweden. The Crime Register includes no offenses committed before this age, and appeals and higher court decisions are not coded. Plea-bargaining is not allowed, which removes the risk of having charges for violent crimes pleaded down and recorded as convictions for nonviolent crimes.

The Total Population Register (Statistics Sweden) provided information on sex, birth year, and parents’ country of birth, since 1932.

The Cause of Death Registry and Migration Registers (Statistics Sweden) contain information on the cause of death in the Swedish population, 1961–2004. They are updated annually according to WHO’s diagnostic system ICD-8/ICD-9 and ICD-10, and were used to verify whether individuals were alive and residing in Sweden during the follow-up period.

The Compulsory 9-year Comprehensive School Register provided data on grades from the final compulsory school year, from 1988 to 1997.

The Hospital Discharge Register (1973–2004, National Board of Health and Welfare) provided information on psychiatric disorders at the time of discharge from the hospital, according to the WHO’s ICD-8/ICD-9 (codes 290–319) and ICD-10 (codes F00–F99).

### Measures

#### Violent crime

Violent crime (including attempted and aggravated forms when applicable) was defined as any of the following: homicide, manslaughter, assault, robbery, threats, and violence against an officer, gross violation of a woman’s or an individual’s integrity, unlawful coercion, unlawful threat, kidnapping, illegal confinement, arson, and intimidation. Sexual offenses may have a partially different etiology [27–29] and were excluded from the analyses. Cases were defined as persons having ever been convicted of a violent crime. Aggravated violent crime was defined as murder, manslaughter, aggravated assault and aggravated robbery. Data were obtained from the Crime Register.

#### Risk factors

All risk factors were analyzed for the whole follow-up period and are described below and in Table 2:

1. From the Hospital Discharge Register, we identified: major mental disorders, such as schizophrenia (ICD-8: 295.0–295.6 or 295.8–295.9; ICD-9: 295A–295F, 295G, 295W–295X; ICD-10: F20) and bipolar disorder (ICD-8: 296.1, 296.3, 296.8; ICD-9: 296A, 296C–296E or 296W; ICD-10: F30–F31); substance use disorders [ICD-8: 304; ICD-9: 304 or 305X; ICD-10: F110–F114, F116–F119, F190–F194 or F196–F199 (i.e., F11–F19 ex. x.5)], and (ICD-8: 303; ICD-9: 303, 305A; ICD-10: F100–F104 or F106–F109), which refer to abuse of and/or dependence on alcohol, narcotics, and/or other illegal substances; and personality disorders (ICD-8: 301; ICD-9: 301; ICD-10: F60–F62).
2. Age at first conviction for a violent crime, based on the earliest date for which an individual was convicted for a violent crime as previously defined, was obtained from the Crime Register. These data were used to subdivide offenders into three separate subgroups based on age at first offense (15–18 years, 19–23 years, and 24 years and older).
3. Data on missing school grades showing academic performance, defined as any or no missing grades out of 16 at the end of the final compulsory school year, were obtained from the Compulsory 9-year Comprehensive School Register. Missing an entire academic performance grade in school indicates substantial truancy and carries with it a more severe consequence for the student than a failing grade.
4. Three nonviolent crime categories were retrieved from the Crime Register and classified as “Theft” (petty theft, grand larceny, grand theft auto), “Drug-related Offense” (transporting, smuggling, storing, selling, producing, and, from 1988, personal use of illegal narcotic substances), and “Traffic Violation” (reckless endangerment, driving under the influence, fleeing the scene of an accident), and were used both separately and together in the single category, “Nonviolent Crime Conviction.”
5. Information on whether a parent had any conviction for a violent or nonviolent crime within the follow-up period or prior to 1973 (during the lifetime of the person identified as a case) was collected from the Crime Register.
6. Parental nationality was collected from the Total Population Register and indexed as the case having one or both parents born outside Scandinavia.
7. Any psychiatric inpatient diagnosis in a parent (not including substance use disorders) was classified as ICD-8: 290–315; ICD-9: 290–319; or ICD-10: F00–F99. Parental substance and alcohol abuse were characterized by the following ICD-codes: ICD-8: 303, 304; ICD-9: 303, 304, 305A, 305 X; ICD-10: F100–F104 or F106–F109, F110–F114, F116–F119, F190–F194, or F196–F199 (i.e., F11–F19 excluding. x.5).

### Subjects

**Fig. 1:**
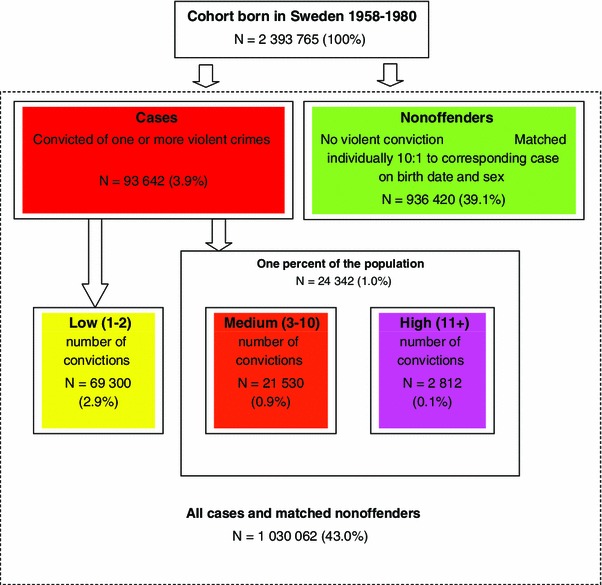
A flowchart of subdivisions of the population

We identified 23,93,765 individuals born in Sweden who, at age 15 (between 1973 and 2004), resided in Sweden, and who were at least 24 years of age at the end of 2004 (Fig. 1). No first-generation immigrants were included in this study, due to missing and/or incomplete data. Of this cohort, we selected all 93,642 violent offenders, defined as those with one or more convictions for a violent crime. In addition, for each violent offender, 10 non-offenders—individuals not convicted for violent crimes between 1973 and 2004—were matched for sex, birth year and month, and for having a sibling of the same age and sex as the case and used for comparison. Non-offenders were then randomly selected from this cohort, giving a total of 9,36,420 non-offenders and a final study population of 10,30,062 for the statistical analyses of risk factors for persistence in violence.

The common practice of selecting four controls to a case is based on the estimation that the power is only slightly increased for each additional control beyond that number. However, this estimation is based on the assumption that no matching has been done and that no further statistical adjustments will be made. If matching or other adjustments are made, more controls are needed to ensure the same power. Regardless of matching, each additional control will indeed always increase power, if only slightly. In the present study, we deemed that ten controls gave us maximal power without unnecessarily increasing computational time.

### Statistical analyses

Initially, the distribution of violent crime convictions was plotted in a frequency table (to assess the proportion of offenders responsible for >50 % of all crimes). From this plot, it was clear that the distribution was highly skewed, with a screen at about 1 % of the population who had three or more convictions and a high top in the most violent 0.1 %. Based on the plot, violent offenders were subsequently divided into three separate subgroups: low persistence, comprising all individuals with one or two convictions for violent crimes; medium persistence, with 3–10 convictions; and high persistence, with 11 or more convictions (Fig. 1). Based on the plot, gender effects and the risk of reconviction by each additional conviction were assessed. In the first step of statistical analyses, repeated Chi-square tests were used to compare possible risk factors across the three violent offender subgroups and the non-offenders (Table 1). In the second step, each factor that differed significantly between low persistence and medium-to-high persistence in the univariate comparisons was included in a multivariate logistic regression model to identify the most important risk factors for persistence in violent crime (Table 3).

**Table 1:** Characteristics of individuals born in Sweden 1958–1980, by number of convictions for violent crimes 1973–2004

Characteristics	Persistence in violent crime expressed by number of convictions							
	High persistence (11+) (*n* = 2,812)	Percent within group (%)	Medium persistence (3–10) (*n* = 21,530)	Percent within group (%)	Low persistence (1–2) (*n* = 6,900)	Percent within group (%)	Non-offenders (0) (*n* = 9,36,420)	Percent within group (%)
Sex								
Male	2,752	97.9	20,214	93.9	60,558	87.4	8,35,240	89.2
Female	60	2.1	1,316	6.1	8,742	12.6	1,01,180	10.8
Any missing school grade	243	8.6	1,720	8.0	3,003	4.3	9,118	1.0
Age at 1st conviction for a violent crime:								
15–18 years	1,691	60.0	9,095	42.0	18,862	27.0	N/A	N/A
19–23 years	773	27.0	7,065	33.0	25,341	37.0	N/A	N/A
≥24 years	348	12.0	5,370	25.0	25,097	36.0	N/A	N/A
Sum of convictions for violent crimes	46,401	–	1,01,767	–	86,215	–	N/A	N/A
Mean violent convictions/individual	16.5	–	4.7	–	1.2	–	N/A	N/A
Number convicted of aggravated violence	2,769	98.5	19,569	90.9	48,437	51.7	N/A	N/A
Sum of convictions for aggravated violence	23,362	–	58,335	–	55,665	–	N/A	N/A
Mean aggravated violence convictions/individual	8.3	–	2.7	–	0.8	–	N/A	N/A
Number convicted of nonviolent crime (any below)	2,746	97.7	18,240	84.7	41,134	59.4	1,83,792	19.6
Theft	2,607	92.7	15,214	70.7	28,542	41.2	82,976	8.9
Drug-related offense	1,905	67.7	7,993	37.1	9,724	14.0	13,839	1.5
Traffic violation	2,440	86.8	14,243	66.2	28,741	41.5	1,29,785	13.9
Sum of convictions for other crimes	1,42,525	–	3,92,211	–	3,60,144	–	5,17,660	–
Mean of other crime convictions/individual	50.7	–	18.2	–	5.2	–	0.6	–
Any psychiatric inpatient diagnosis:	1,778	63.2	7,512	34.9	10,367	15.0	21,404	2.3
Major mental disorder	157	5.6	784	3.6	1,438	2.1	4,749	0.5
Personality disorder	624	22.2	1,902	8.8	2,228	3.2	3,859	0.4
Substance use disorder	1,679	59.7	6,773	31.5	8,777	12.7	15,407	1.6
One or both parents of cases and non-offenders								
Non-Scandinavian ethnicity	371	13.2	2,804	13.0	7,329	10.6	65,656	7.0
Any conviction for a violent crime	699	24.9	3,911	18.2	8,057	11.6	31,555	3.4
Any conviction for a nonviolent crime	1,850	65.8	12,681	58.9	34,397	49.6	2,78,204	30.7
Any psychiatric inpatient diagnosis (not including substance use disorder)	1,350	48.0	8,398	39.0	21,296	30.7	1,63,804	17.5
Any inpatient diagnosis substance use disorder	941	33.5	5,847	27.2	14,089	20.3	98,382	11.5
Parent died before child was 18 years old	295	10.5	1,834	8.5	4,963	7.2	43,544	5.7

All comparisons yielded significant differences across all groups at *P* < 0.001 (Chi-square test)

The fact that the youngest individuals in the cohort had a shorter follow-up period, and therefore, a shorter time at risk, was taken into consideration by calculating the relative proportions of low-, versus medium- and high-persistence offenders by each birth year. We found that the proportion of offender groups was highly consistent in each cohort and differed by less than 7 % for subjects born between 1958 and 1980 (Appendix Table 4). According to this calculation, it is possible that another 100–150 of the youngest cases (with the shortest follow-up periods) went on to relapse after 2004. However, an underestimation of relapses of this size, as compared to the total group size of the medium- and high-persistence offenders (which encompassed over 24,000 individuals), would have no or very little impact on the findings presented on the distribution of violent crimes. In the subsequent analyses of risk factors, the use of age-matched non-offender controls prevents any bias from the differences in follow-up periods in offenders of different ages.

Statistical analyses were performed with statistics software Predictive Analytics Software, version 18.

## Results

### Descriptives

#### Distribution of violent convictions

**Fig. 2:**
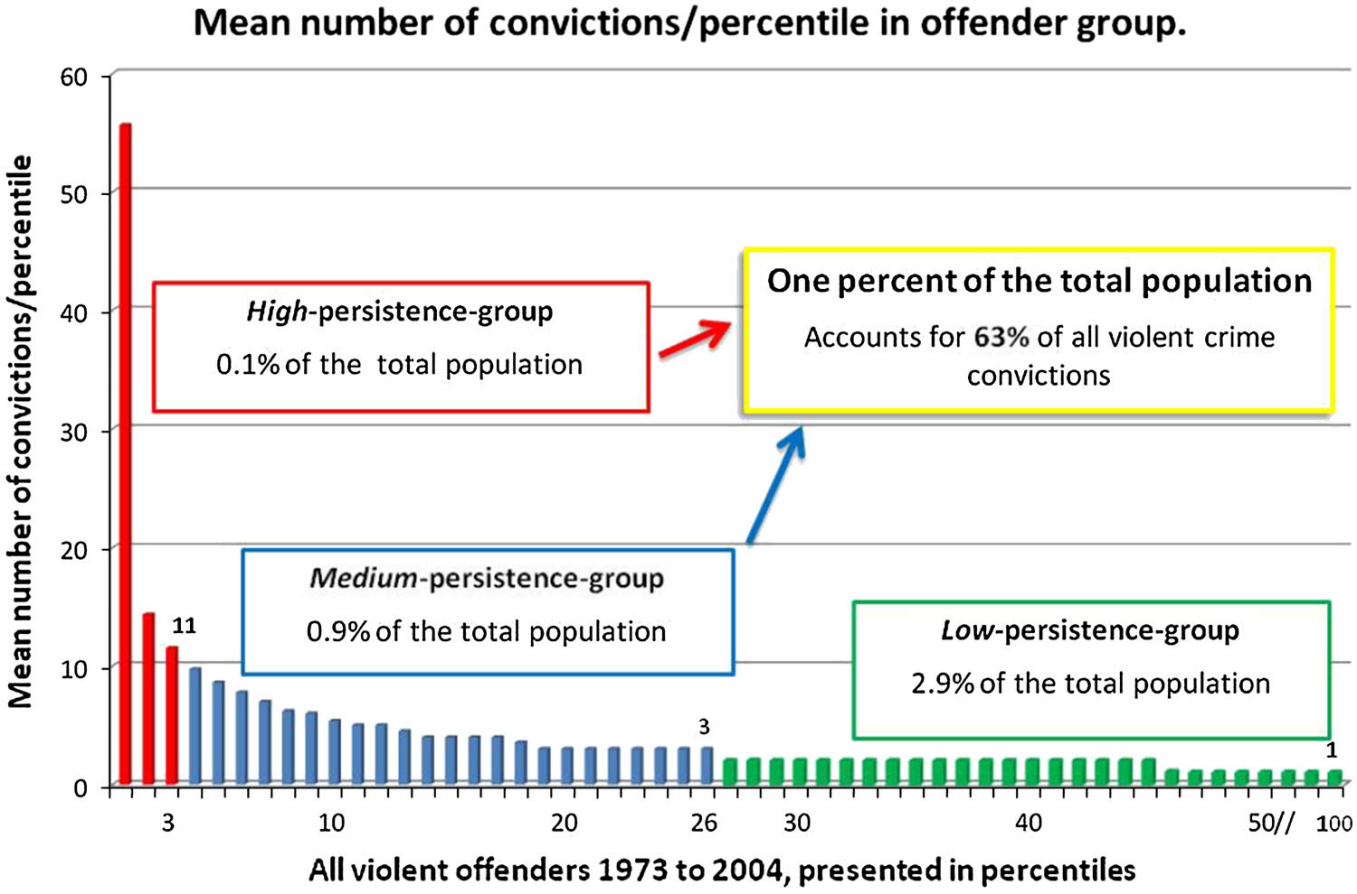
Distribution of violent crime convictions by percentiles in the total offender group (3.9 % of the total population, *n*: 93,642) in the follow-up period from 1973 to 2004. Each percentile equals 936 violent crime offenders

A total of 93,642 individuals (3.9 % of the total population cohort) were convicted of one or more violent crimes (Figs. 1, 2, referred to as the “offender group”). Of these, 83,524 (89.2 %) were men. The total number of convictions for violent crimes between 1973 and 2004 was 2,34,383, ranging from 1 to 80 per individual (mean 2.5, SD = 3.3, median 1). The distribution of violent crime convictions is detailed graphically by percentiles of the offender group in Fig. 2, showing that 1 % of the population accounted for the majority (more than 50 %) of all violent convictions, with a cut-off at three or more convictions. A high top of the mean number of convictions per individual was also well identified, with a cut-off at 11 or more convictions. Therefore, three groups of offenders were created: low-, medium-, and high-persistence offenders.

Low-persistence offenders (*n* = 69,300, 1 or 2 violent crime convictions per individual) constituted 2.9 % of the total population cohort and 74.0 % of all individuals ever convicted of a violent crime (i.e. the offender group). They accounted for 36.8 % of all violent crime convictions during the study period (86,215 convictions; including 55,665 for aggravated violence; i.e., 40.5 % of all aggravated violent convictions).

Medium-persistence offenders (*n* = 21,530, 3–10 violent crime convictions per individual) constituted 0.9 % of the total population cohort and 23.0 % of the offender group. They accounted for 43.4 % of all violent crime convictions (1,01,767 convictions; including 57,335 for aggravated violence; i.e., 42.5 % of all aggravated violent convictions).

High-persistence offenders (*n* = 2,812, 11 or more violent crime convictions per individual) constituted 0.1 % of the total population cohort and 3.0 % of the offender group. They accounted for 19.8 % of all violent crime convictions (46,401 convictions, including 23,362 for aggravated violence; i.e., 17.0 % of all aggravated violent convictions).

Together, the medium- and high-persistence violent offenders (*n* = 24,342, with 3 or more violent crime convictions per individual) constituted 1.0 % of the total population cohort and 26.0 % of the offender group. They accounted for 63.2 % of all violent crime convictions during the study period (mean 6.0, SD = 4.8, median 4, a total of 148,168 convictions; including 81,967 for aggravated violence; i.e., 59.5 % of all aggravated violent convictions).

#### Gender differences

Overall, women accounted for 10.8 % of violent offenders, but the proportion of female offenders decreased with the number of violent convictions per individual, from 12.6 % in the low-persistence group to 6.1 % in the medium-persistence group and 2.1 % in the high-persistence group. The male/female skew was also more pronounced for aggravated violent crimes than for all violent crimes. Women accounted for 6,286 (11 %) of those convicted of 1 or 2 aggravated violent crimes, 499 (3.8 %) of those with 3–10 aggravated violent crime convictions, and 7 (1.0 %) of those with more than 10 convictions for aggravated violent crimes. The median age at the first conviction for any violent crime was 22.0 years (SD = 7.2) for women and 21.0 years (SD = 6.1) for men (*t* = 19.0, *P* < 0.001).

#### Persistence in violent crime

Table 2 and Fig. 2 present the accumulated number of violent crime convictions per individual, according to the level of persistence, together with the number and proportion of convictions that could be prevented, were it possible to halt violent crime careers after a specific number of convictions.

**Table 2:** Number of individuals and number of convictions for violent crimes, 1973–2004, among all individuals born in Sweden 1958–1980

Individuals				Convictions			
Number of violent convictions^a^	Number individuals/number of conviction	Cumulative number of individuals^b^	Cumulative percent of the total population^c^(%)	Cumulative number of violent convictions^d^	Percent of all violent convictions (%)	Preventive potential of all convictions^e^	
1	52,385	93,642	3.91	2,34,383	100	181,998	78 %
2	16,915	41,257	1.72	1,81,998		148,168	63 %
3	7,907	24,342	**1.02**	1,48,168	**63**	124,447	**53** **%**
4	4,739	16,435	0.69	1,24,447		105,491	45 %
5	2,923	11,696	0.49	1,05,491		90,876	39 %
6	2,018	8,773	0.37	90,876		78,768	34 %
7	1,355	6,755	0.28	78,768		69,283	30 %
8	1,085	5,400	0.23	69,283		60,603	26 %
9	828	4,315	0.18	60,603		53,151	23 %
10	675	3,487	0.15	53,151		46,401	20 %
11	492	2,812	**0.12**	46,401	**20**	40,989	**17** **%**
12	396	2,320	0.10	40,989		36,237	15 %
13	312	1,924	0.08	36,237		32,181	14 %
14	275	1,612	0.07	32,181		28,331	12 %
15	207	1,337	0.06	28,331		25,226	11 %
16	196	1,130	0.05	25,226		22,090	9 %
17	135	934	0.04	22,090		19,795	8 %
18	132	799	0.03	19,795		17,419	7 %
19	82	667	0.03	17,419		15,861	7 %
20	77	585	0.02	15,861		14,321	6 %
21	60	508	0.02	14,321		13,061	6 %
22	61	448	0.02	13,061		11,719	5 %
23	50	387	0.02	11,719		10,569	5 %
24	46	337	0.01	10,569		9,465	4 %
25–80	291	291	0.01	9,465	4	N/A	N/A

Relevant and important values are indicated in bold

^a^Number of convictions per individual for the whole follow-up period from 1973 to 2004 (level of persistence)

^b^Accumulated number of individuals with the number of convictions or higher, provided in the first column

^c^Percent of population with the number of convictions or higher, provided in the first column

^d^Accumulated number of violent convictions accounted for by violent offenders with the number of convictions or higher, per individual, provided in the first column

^e^Number and percentage of violent convictions 1973–2004 that could have been avoided if offenders, with the number of convictions or higher per individual, provided in the first column, had been prevented from recidivating

Among individuals convicted of one violent crime, 44 % (41,257 of 93,642) were reconvicted. After 2 violent convictions, 59 % (24,342 of 41,257) were reconvicted, and after 3 convictions, 68 % (16,435 of 24,342) were reconvicted. If violent careers could be stopped after 3 convictions, 53 % of all violent convictions would be prevented. The recurrence rate increased from about 70 % after 4 convictions to about 80 % after 7 and to about 90 % after 11 crimes per individual, after which the low number of perpetrators at each new step made further analyses difficult to interpret.

### Risk factors for persistence in violent crime

#### Criminal history, mental health, and educational background

Data on criminal history, mental health, and parental characteristics are presented in Table 1. All examined variables differed significantly across the four subgroups (*P* < 0.001, Chi-square tests). The proportion of aggravated violent crimes and the number of convictions for nonviolent crimes increased substantially with increasing persistence of violent convictions, as did the proportion of subjects with an early onset of violent crime (convictions before the age of 19) and school failure. Although the proportion of subjects diagnosed with mental health problems increased with the number of violent convictions, doubling from the low-persistence to the high-persistence group, personality and substance use disorders increased between 5 and 7 times. Perpetrators of aggravated violence had a somewhat lower prevalence of major mental disorders (2.8 %) than perpetrators of any violent crime (3.7 %).

#### Parental risk factors

All parental factors differed significantly (*P* < 0.001 by Chi-square test) across groups (Table 1): 48.0 % of high-persistence offenders had a parent with a psychiatric disorder, compared with 30.7 % of low-persistence offenders and 17.5 % of non-offenders. Moreover, 24.9 % of high-persistence offenders had a parent convicted of a violent crime, compared to 11.6 % of low-persistence offenders and 3.4 % of non-offenders. Finally, 65.8 % of high-persistence offenders had a parent convicted of a nonviolent crime compared to 49.6 % of low-persistence offenders and 30.7 % of non-offenders.

#### Multivariate analyses of risk factors for persistence in violent crime

A multiple logistic regression was used to model 15 dichotomous risk factors for medium and high persistence in violence (i.e., 3 or more violent crime convictions) versus low persistence (Table 3). Male sex, personality disorders, and a first conviction for violence between the ages of 15 and 18 had the highest ORs (2.0–2.5), followed by substance-related problems (“Any conviction for a drug-related offence” and “Any diagnosis of substance use disorder”), with OR 1.9. All parental factors, except “Parent with any conviction for a violent crime” (OR 1.3), had ORs close to 1.

**Table 3:** Multivariate logistic regression model of risk factors for the persistent 1 % of the total population with 3 or more convictions for violent crime 1973–2004, compared to the low-persistence group

Predictor	*B*	Odds ratio	95 % CI
Male sex	0.9	2.5^a^	2.3–2.6
Any missing school grade	0.2	1.3^a^	1.2–1.4
1st conviction for violence, age 15–18	0.7	2.0^a^	1.9–2.0
Any conviction for theft	0.7	2.0^a^	1.9–2.0
Any conviction for a drug-related offense	0.7	1.9^a^	1.9–2.0
Any conviction for a traffic violation	0.6	1.8^a^	1.7–1.8
Any diagnosis of a major mental disorder	0.2	1.3^a^	1.1–1.4
Any diagnosis of a personality disorder	0.8	2.3^a^	2.1–2.4
Any diagnosis of a substance use disorder	0.6	1.9^a^	1.8–2.0
Parent of non-Scandinavian ethnicity	0.1	1.1^a^	1.0–1.1
Parent with any conviction of a violent crime	0.3	1.3^a^	1.2–1.4
Parent with any conviction of a nonviolent crime	0.1	1.1^a^	1.1–1.1
Parent diagnosed with a psychiatric disorder	0.1	1.1^a^	1.1–1.1
Parent diagnosed with a substance use disorder		–	
Parent died before child’s 18th birthday	0.1	1.1^b^	1.0–1.1

^a^Odds ratios significant at *P* < 0.001

^b^Odds ratios significant at *P* < 0.05

A model excluding women from the analyses yielded only a few minor differences (Appendix Table 5) and did not change the overall pattern of risk factors.

## Discussion

### Generalizability

This study used merged Swedish nationwide longitudinal registers, including data on parents and criminal convictions, over a span of 32 years, to describe the distribution of violent crime in the total population and personal risk factors for individual persistence in violence among the offenders. Since these levels of violent crime and their judicial resolution (case clearance) are similar to those in most countries in the western EU and in Canada [30], our results for prevalence and distribution could be generalizable within this cultural sphere, while the identified risk factors for persistence in especially violence-prone individuals are probably more widely generalizable across cultures. Socioeconomic factors would most certainly have proved to be important had they been included in the analyses, but they were excluded in this study in favor of individual risk factors to focus on the relation between potentially treatable or predictive mental health factors and persistence in violence. Further investigations of the relationship between socioeconomic factors and the individual risk factors considered here are clearly warranted. Separate studies of individual risk factors and of class or socioeconomic factors will have to be made side by side and interpreted for the different purposes of individual treatment or prevention versus societal efforts.

### Comments to main findings

In summary, we confirmed the important parental and psychiatric risk factors for any violent crime suggested in prior research primarily based on clinical samples [31] and in a small body of population-based, epidemiological studies [9, 15, 32]. In relation to the persistence of violent crime (defined by the number of violent convictions per individual), we identified male sex, early onset of violent criminality, personality disorders, and substance use disorders as the most important risk factors specific to persistence in violence.

#### Distribution and preventive potential

About one quarter of all those who were ever convicted of a violent crime (i.e., the offender group), corresponding to the most persistently violent 1 % of the study population, were responsible for a total of 63 % of all violent crime convictions in the country, while almost three quarters of all violent offenders, corresponding to 2.9 % of the population, were convicted only once or twice during the study period. This is in line with previous research that shows a small group of criminally active young adult men to be responsible for a large part of all violent crimes [3, 4, 33] and the high impact of a very small group of especially violent individuals indicated by the high reconviction rates among former prison detainees [8]. The numbers (and relative proportions) of violent convictions that theoretically could be prevented if individuals were stopped from committing further crimes at specific stages of relapsing into violence may be used to plan preventive strategies against violent crime (Table 2). If all violent crime careers could come to a stop after a third conviction (which would require interventions directed at 1 % of the total population), more than 50 % of all convictions for violent crime in the total population would be prevented.

#### Gender aspects

The fact that females represented a mere 11 % of all those convicted and 6 % of the persistently violent concurs with previous findings. When considering persistence in violent crime and convictions for aggravated violent crimes, females represented an even smaller part of the group. The female group was too small for detailed subgroup analyses of risk factors. Other studies have reported more complex and severe mental health problems, including higher frequencies of combinations of substance use disorders, personality disorders, major mental disorders and other susceptibility factors in female offender groups [34].

#### Risk factors, prevention, and treatment strategies

First offenses are particularly difficult to predict, especially due to the low base rates of violent crime overall. By contrast, the majority of violent crimes are committed by a group of offenders who may be identified by rather easily observable features, such as having already been convicted of violent crimes several times already in adolescence, and having problems with substance abuse.

These statistics seemingly support the catchphrase and model employed in California and several other states in the USA, “three strikes and you’re out.” This model, however, does not seem to have been as successful as was hoped when it was initiated in the mid-1990s [35, 36]. The “three strikes law” was intended to act as a deterrent and keep offenders from committing aggravated and violent crimes, but its effect is difficult to ascertain; overall prevalence of crime has remained quite stable in the USA, while the prison population has increased to quite dramatic levels in some places and in some social groups [37]. One explanation could be that the “three strikes law” also applies to nonviolent crimes, thus creating too large a group of detainees to be given sufficient shares of scarce treatment and rehabilitation resources. Proponents, however, have argued that a major increase in criminality may have been prevented through the “three strikes law” [35]. The group identified here as medium- or high-persistent violent offenders, those with three or more convictions, could be small enough to single out for more costly treatment efforts for their often clear-cut psychosocial and mental health needs—efforts that might also potentially yield a substantial reduction in overall societal violence. Ethically, it is easier to defend treatment efforts that reduce autonomy for people who have actually committed a number of violent crimes than for everyone considered likely to do so in the future. If a “three strikes” policy were applied only to cases of violent crime rather than to all types of crime, a smaller group of individuals would be targeted and could therefore be given more resource-intensive treatment and preventive efforts. Specific prevention of violent recidivism may include detention or intensive surveillance programs including electronic surveillance, intensive care management, and monitoring for drug and alcohol use. But a wide array of other treatment and support efforts, such as education, work training, housing, mental and somatic health care, psychotherapy, and other social care interventions could also be used. For a detailed and current overview of the field of treatments for violent propensities, see Raine [38]. The cut-off point of three convictions may also work as a simple pedagogic tool for more effective preventive efforts, as it is easily explained to adolescents and includes two opportunities for increasingly severe “warnings” to a young person on the path of violence.

Based on the results of this and previous studies [3, 9], it should be possible to identify individuals most at risk for developing persistent violent criminal behavior as early as adolescence, since an early onset in violent criminality manifested through convictions for violent crimes seems to be one of the strongest predictors of persistence in violence later in life [3]. Therefore, developing and improving preventive strategies for delinquent youth who display violent tendencies should be a priority. Here, as in the overall prevention of violence in society, political efforts are important, since rates of early engagement in violent behavior may be highly dependent on sociocultural factors, which are malleable and may be ameliorated through political effort [39]. Specific efforts targeting the group of especially violent individuals described in this study, along with preventive efforts aimed at youth, may provide a complimentary inroad to achieve a significant reduction in overall levels of violent crime.

Further research to establish evidence-based and cost-effective preventive methods against violence is obviously necessary. Currently, in many penal systems, including the Swedish, many resources are invested in preventing those diagnosed with major mental disorders from relapsing into criminal behaviors, even if their overall contribution to violence is small (3.7 % of all convictions) and only moderately linked to persistence in violence. Several other studies have also shown that people with major mental disorders share the same risk factors for persistence in violent crime as others who have been convicted of violent crimes, especially when substance abuse is involved [31, 40]. Based on the current results, what is needed are research efforts focused on developing suitable preventive measures that specifically address the risk factors with the strongest contribution to persistence in violence.

### Limitations

The skewness in the data may, to some extent, be context specific and could be less extreme in societies with a higher base prevalence of violent crime than Sweden. The fact that socioeconomic factors were not included in this study prevents analyzes the importance of adverse social circumstances to the development of persistent violent behavior. Nevertheless, the results suggest that even in a country such as Sweden, with a long tradition of tax-funded general health and social welfare policies, a very few individuals do repeatedly commit violent offenses and may reach tens of convictions for severe crimes during a study period of just over 2 decades. Importantly, they continue to do so despite negative attitudes in the general population against the use of violence, and despite penal sanctions (including loss of freedom) and other current preventive or treatment efforts [41].

Since the analyses of mental disorders presented here are based only on inpatient diagnoses from the Hospital Discharge Register, it is likely that we underestimated the actual prevalence of these disorders, especially personality disorders and substance use disorders. The marked difference in these characteristics between the offenders and the non-offenders, however, could represent a difference in the severity of these disorders, rather than differences in actual group prevalences. Another limitation of the analyses of personality disorders in this study is that this category includes all types of personality disorders. Therefore, it is possible that the increased risk detected was mitigated by the admixture of personality disorders that actually reduce the risk of violence.

As this study was cross-sectional and the only dates included in the current data file were for year and month of birth and year and month of first conviction for a violent crime, the exact order between risk factors and violent crime could not be teased out. For instance, it is possible that some subjects were given their psychiatric diagnoses after their first convictions. Studies focusing on the causality between psychotic disorders and criminality have shown an increased risk for homicide in the primary stages of the disorder (before diagnosis and treatment) [42]. Moreover, a recently published study by Coid et al. [43] may show important associations between anger-related delusions and both minor and serious violence, even in the year prior to first contact with health care. Therefore, our results on the role of major mental disorders in violence should be interpreted with caution. On the other hand, many of the psychiatric diagnoses are considered life-long, with problems arising early, and the results that we present in relation to persistence in violence concur with numerous case–control studies [31]. Since no data on behavior, mental health, or criminal acts before the age of criminal responsibility are available, we did not have the opportunity in this study to consider or discuss the specific pathway of life-course persistent antisocial behavior.

In addition, one might question our use of the number of violent convictions, rather than the actual number of crimes per individual—the so-called “dark figure” of crime—retrieved from self-reports or collateral interviews. This “dark figure” haunts many types of research on crime, but as no accurate measures of unreported and/or unconvicted crimes are available, it seems reasonable to assume that official data on crime are the tip of the iceberg and thereby provide a crude estimate of what is below the surface. In support of this, different indices of violent crime are highly related, and different sources of information alter the actual base rates but not the strength of association between variables [12].

This study did not include data on sentences carried out, so it is possible that some of those convicted once or twice were given long prison sentences, and were thereby prevented from committing additional crimes. Almost 60 % of those convicted once or twice included an aggravated violent crime, which carries 4 years to life imprisonment. Further investigation is needed to address the effect of detainment, even if studies of violence also have to consider periods in the penal system, where new violent crimes are not especially rare [20].

As this study did not consider socioeconomic factors, and because of the great differences in law and efficacy of law enforcement globally, the generalizability of the criminological results presented might be restricted to the countries in the western EU and to Canada.

## Conclusion

The vast majority of violent crimes are perpetrated by a small number of persistent violent offenders, almost all males, who have an early onset of violent criminality and display substance use problems, personality disorders, and nonviolent criminality. These findings support the provision of far-reaching interventions among young individuals who have committed one or two violent crimes and are at risk of developing persistent violent criminal behavior.

## Appendix

See Appendix Tables 4, 5.

**Table 4:** The distribution of the number of convictions by birth year and the number of individuals convicted of a violent crime. (*N*: 93,642)

	1958	1959	1960	1961	1962	1963	1964	1965	1966
Low^a^	**71.0** **%**	**71.2** **%**	**72.2** **%**	**71.4** **%**	**73.0** **%**	**73.4** **%**	**73.6** **%**	**72.8** **%**	**74.9** **%**
Medium^b^	24.6 %	24.2 %	24.2 %	24.5 %	23.6 %	23.0 %	23.0 %	23.8 %	22.5 %
High^c^	4.4 %	4.6 %	3.6 %	4.0 %	3.5 %	3.5 %	3.4 %	3.4 %	2.6 %
*n*	4,893	4,716	4,451	4,612	4,699	4,814	5,018	4,973	4,770

	1967	1968	1969	1970	1971	1972	1973	1974	1975
Low^a^	**74.2** **%**	**76.6** **%**	**74.8** **%**	**74.4** **%**	**76.7** **%**	**75.7** **%**	**74.8** **%**	**74.5** **%**	**74.5** **%**
Medium^b^	22.9 %	20.8 %	22.3 %	22.9 %	21.0 %	21.8 %	22.6 %	22.7 %	23.5 %
High^c^	2.9 %	2.6 %	2.9 %	2.7 %	2.3 %	2.5 %	2.6 %	2.9 %	1.9 %
*n*	4,651	4,088	3,812	3,879	3,969	3,685	3,590	3,712	3,414

	1976	1977	1978	1979	1980				
Low^a^	**74.7** **%**	**74.9** **%**	**74.7** **%**	**74.9** **%**	**77.5** **%**				
Medium^b^	22.9 %	22.8 %	23.4 %	23.2 %	20.6 %				
High^c^	2.4 %	2.3 %	1.9 %	2.0 %	1.9 %				
*n*	3,300	3,302	3,217	3,156	2,921				

Relevant and important values are indicated in bold

^a^1–2 convictions for violent crimes

^b^3–10 convictions for violent crimes

^c^11 or more convictions for violent crimes

**Table 5:** Multivariate logistic regression model of risk factors for the persistent 1 % of the total population with 3 or more convictions for violent crime from 1973 to 2004 (women excluded), compared to the low-persistence group

Predictor	*B*	Odds ratio	95 % CI
Any missing school grade	0.2	1.3^a^	1.2–1.4
1st conviction for violence at age 15–18 years	0.7	2.0^a^	1.9–2.0
Any conviction for theft	0.7	2.0^a^	1.9–2.0
Any conviction for a drug-related offense	0.7	2.0^a^	1.9–2.1
Any conviction for a traffic violation	0.6	1.8^a^	1.7–1.9
Any diagnosis of a major mental disorder	0.2	1.2^b^	1.0–1.3
Any diagnosis of a personality disorder	0.8	2.3^a^	2.1–2.4
Any diagnosis of a substance use disorder	0.7	1.9^a^	1.9–2.0
Parent of non-Scandinavian ethnicity	0.1	1.1^a^	1.0–1.1
Parent with any conviction for a violent crime	0.3	1.3^a^	1.2–1.4
Parent with any conviction for a nonviolent crime	0.1	1.1^a^	1.1–1.1
Parent diagnosed with a psychiatric disorder	0.1	1.1^a^	1.1–1.2
Parent diagnosed with a substance use disorder		–	
Parent who died before child’s 18th birthday	0.1	1.1^b^	1.0–1.1

^a^Odds ratios significant at *P* < 0.001

^b^Odds ratios significant at *P* < 0.05
